# Impact of lifestyle and environmental factors on fertility

**DOI:** 10.1097/MOU.0000000000001339

**Published:** 2025-09-15

**Authors:** Anett Szabó, Péter Nyirády, Zsolt Kopa

**Affiliations:** a Department of Urology; b Centre for Translational Medicine, Semmelweis University, Budapest, Hungary

**Keywords:** DNA fragmentation index, environmental exposures, modifiable risk factors, subfertility

## Abstract

**Purpose of review:**

Infertility affects approximately 15% of couples, with male factors implicated in more than 50% of cases. Concerns over declining semen quality – evidenced by a more than 50% drop in sperm concentration over four decades – have triggered investigation into modifiable lifestyle and environmental factors. This review summarizes recent evidence on exposures that negatively impact male fertility.

**Recent findings:**

Smoking increases sperm DNA fragmentation (SDF) by approximately 10% and alters hormonal profiles; e-cigarettes may carry similar risks. Chronic alcohol use raises SDF by a comparable magnitude, disrupts the hypothalamic–pituitary–gonadal axis, and may cause testicular atrophy. Obesity impairs spermatogenesis through aromatase-mediated hormonal imbalance and inflammation; and even modest weight loss improves sperm parameters. Drug use – particularly steroids, cannabis, and opioids – may also suppress fertility. Environmental exposures such as heat waves, fine particulate matter, and endocrine-disrupting chemicals reduce semen quality and can contribute to testicular dysgenesis. Conversely, adherence to certain diets, antioxidant supplementation modestly improves sperm quality and reduce SDF.

**Summary:**

Lifestyle and environmental factors significantly impair male reproductive health through hormonal disruption, oxidative stress, and direct germ cell damage. These risks are common and often reversible. Identifying and modifying such exposures is essential for improving fertility outcomes and reducing long-term health burdens.

## INTRODUCTION

Infertility, defined as the inability to conceive within 12  months of regular unprotected intercourse, affects approximately 15% of couples in industrialized nations [1–3]. Male factors are implicated in up to half of these cases, and the prevalence of male infertility in the general population is estimated to be as high as 15% [4]. The clinical and social consequences of infertility are profound – affecting psychological well being, increasing demand for medically assisted reproductive technologies (MAR), and contributing to a growing public health burden [5]. Moreover, male infertility has been associated with increased risks of morbidity and mortality from cardiovascular, metabolic, autoimmune, and oncologic diseases, possibly due to shared underlying pathophysiological mechanisms [6].

Concerns about a potential decline in male reproductive health were first raised by Carlsen *et al.* [7] in 1992, who published a meta-analysis suggesting a significant decrease in semen volume and sperm concentration over the past five decades. Although the findings remain controversial due to methodological variability, subsequent studies have largely supported this trend, reinforcing concerns about decline in sperm quality worldwide [7–10]. Various hypotheses have been proposed to explain this phenomenon, including genetic and epigenetic inheritance, in-utero exposures, and, evidently, modifiable lifestyle and environmental factors [11].

Recent years have seen renewed scientific interest in the role of environmental and behavioural influences on male fertility, especially following the inclusion of the first evidence-based sperm functional parameter – sperm DNA fragmentation (SDF) – in the international guidelines [12]. This interest is driven by the fact that many risk factors – such as tobacco use, alcohol consumption, poor diet, environmental pollution, and many others – are not only highly prevalent, but also potentially reversible. These factors can adversely impact both classical semen parameters and more advanced markers, such as SDF, chromatin integrity, and epigenetic stability [6]. Their mechanisms of action are multifactorial, involving disruption of the hypothalamic–pituitary–gonadal (HPG) axis, oxidative stress, hormonal dysregulation, and direct damage to testicular cells and the germ line [13].

The growing body of evidence and the urgent need to reduce the personal, social, and economic burden of infertility have made the identification and treatment of modifiable risk factors a top clinical and public health priority. In this review, we summarize the available literature on the impact of lifestyle and environmental exposures on male fertility over the past 18  months. Our aim is to synthesize preventive strategies that can enhance reproductive outcomes and improve long-term male health. 

**Box 1:**
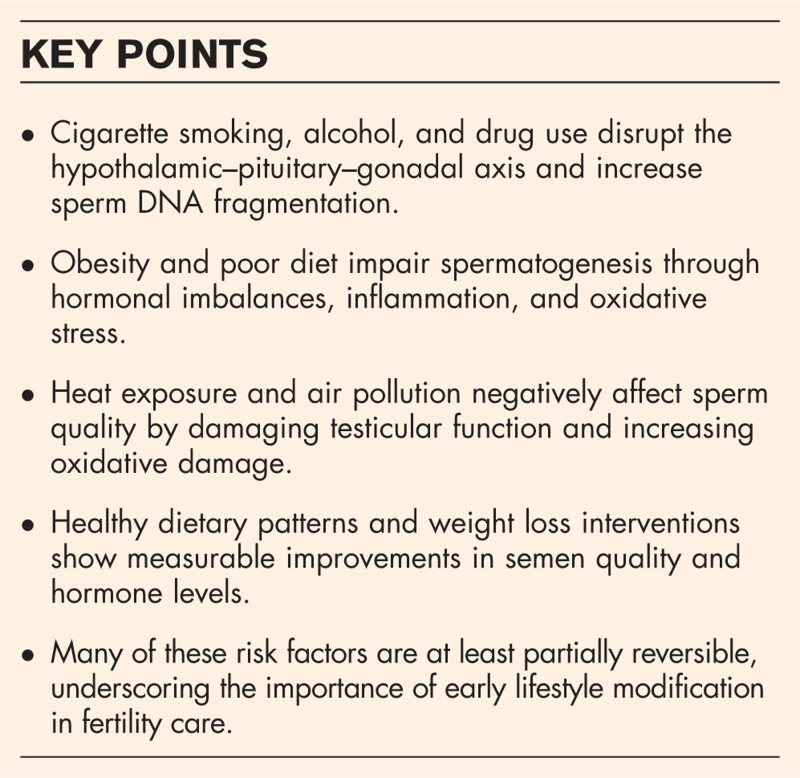
no caption available

## SMOKING

Cigarette smoking has long been recognized as detrimental to male health, including reproductive function. Most studies link smoking to decreased semen quality, such as lower sperm count, reduced motility, and abnormal morphology [14^▪^,15^▪^]. In addition to these conventional parameters, SDF is also more prevalent among smokers, with studies reporting approximately a 10% difference compared to nonsmokers [16^▪▪^]. However, there is a lack of data on the potential reversibility of these effects – specifically, the impact of smoking cessation on SDF remains insufficiently studied.

Hormonal imbalance is also a major consequence of smoking. Heavy smokers (>20 cigarettes/day) show lower testosterone levels, as well as elevated levels of follicle stimulating hormone (FSH) and luteinizing hormone (LH), indicating impaired spermatogenesis and testicular dysfunction [17^▪▪^]. Smoking also reduces seminal zinc levels, thereby increasing oxidative damage [14^▪^].

Although, research into the effects of vaping is still scarce, emerging evidence suggests that e-cigarettes may pose risks similar to conventional smoking. E-liquids contain a mixture of substances – including nicotine and propylene glycol, among others – that each is capable of disrupting testicular homeostasis, either individually or synergistically. Significantly lower sperm concentrations and total sperm counts have been reported in young men who vape daily [18^▪▪^]. Vaping appears to disrupt the HPG axis, altering both hormone secretion and testicular function, such as the mechanism proposed for combustible tobacco [14^▪^]. Nonetheless, due to variable compositions and limited long-term data, the precise extent of e-cigarette-related reproductive harm remains difficult to quantify.

Smoking-related sperm damage is multifactorial, including chromatin defects, oxidative DNA damage, and epigenetic modifications such as aberrant DNA methylation. These changes may compromise embryo development and increase risks of miscarriage and congenital disorders [15^▪^]. Although some improvements in semen quality have been reported after smoking cessation, reversibility is not consistent [17^▪▪^]. A study comparing semen parameters at 3 months before and after smoking cessation found significant improvements in volume, concentration, and total sperm count, although motility and morphology changes were modest [17^▪▪^].

Additionally, polycyclic aromatic hydrocarbons in tobacco smoke have been strongly linked to DNA damage [17^▪▪^]. Smoking affects not only sperm quality but also the testicular microenvironment, reducing Sertoli cell function [15^▪^,^16^^▪▪^]. Recent findings suggest that paternal smoking may contribute to increased risks of childhood diseases, including neurodevelopmental disorders and cancers, through epigenetic inheritance [15^▪^]. Therefore, cessation of both cigarette and e-cigarette use should be prioritized – not only to improve semen parameters and hormonal balance, but also to safeguard long-term reproductive potential and the health of future offspring.

## ALCOHOL

Chronic alcohol consumption exerts a dose-dependent negative effect on male fertility. Ethanol may disrupt the HPG axis, leading to decreased testosterone and altered LH and FSH levels [17^▪▪^,19^▪^]. Heavy drinkers also show testicular atrophy, as well as increased SDF of up to 49.6% compared to nondrinkers exhibiting an SDF of 33.9% [16^▪▪^].

Meta-analyses and cohort studies suggest that alcohol impairs semen volume, sperm concentration, motility, and morphology. Chronic intake of more than 50–60  g/day is consistently associated with reduced semen quality. Binge drinking (having five or more drinks in a row at least once in 2 weeks), even in isolated episodes, can also transiently reduce sperm count and motility, particularly when combined with other lifestyle stressors such as drug use or cigarette smoking [19^▪^].

Although there are no significant adverse effects of occasional alcohol consumption – and in some cases, moderate consumption has been associated with higher probabilities of live birth – the overall trend supports caution [20^▪▪^]. Alcohol alters the production of sex hormone-binding globulin (SHBG) through reducing the bioavailability of testosterone, contributing to hypogonadism. SHBG also appears to have extra-gonadal roles, affecting metabolism and possibly influencing alcohol consumption behaviours through genetic predisposition [19^▪^].

Histological studies on alcoholics show partial or complete arrest of spermatogenesis and a higher incidence of Sertoli cell-only syndrome [19^▪^]. Fortunately, several clinical studies have documented partial or full recovery of spermatogenesis following abstinence from alcohol [16^▪▪^,^17^^▪▪^].

Furthermore, chronic alcohol exposure may indirectly affect fertility through liver dysfunction, nutritional deficiencies, and increased oxidative stress. Impairment of hepatic glycosylation caused by alcohol affects SHBG isoforms, and liver disease can further exacerbate hormonal imbalances [19^▪^].

## OBESITY

Obesity is another significant and growing concern for male fertility. Adipose tissue converts androgens into oestrogens via aromatase activity, resulting in hormonal imbalances that suppress FSH and LH secretion. These imbalances impair spermatogenesis and testosterone production [21^▪^].

Observational and interventional studies confirm the negative impact of obesity on sperm concentration, total count, morphology, and hormonal balance [22^▪^]. In contrast, studies examining the impact of physical activity on SDF are too heterogeneous to draw definitive conclusions. However, in our previous meta-analysis, lifestyle changes were associated with a modest reduction in SDF of −2.94% [95% confidence interval (CI): −4.94 to −0.95] after 3 months [23^▪^].

Lipotoxicity-induced oxidative stress and endoplasmic reticulum dysfunction further deteriorate sperm quality. Obesity also correlates with systemic and metabolic dysfunctions such as insulin resistance and diabetes, both of which further impair sperm production and function. Obese men with concurrent diabetes have the poorest outcomes in terms of sperm motility, morphology, and count [21^▪^].

The adverse effects of obesity on male fertility are multifactorial, extending beyond hormonal disruption and insulin resistance. Obesity is now widely acknowledged as a condition of chronic low-grade inflammation. These pro-inflammatory mediators interfere with Leydig cell function and the integrity of the blood–testis barrier, further impairing spermatogenesis [21^▪^].

Importantly, weight loss interventions show promise in reversing some of these pathophysiological effects. Studies demonstrate that even modest reductions in body weight can lead to significant improvements in testosterone, SHBG, and inhibin B levels, accompanied by improved sperm concentration and morphology [22^▪^].

On the other hand, extreme weight loss is not recommended as being underweight (BMI <18.5 kg/m^2^) can also compromise fertility, prolonging time to pregnancy [24^▪^].

## DRUGS

Anabolic-androgenic steroids (AAS) are known to suppress endogenous gonadotropin secretion and halt spermatogenesis. In their review, Azevedo *et al.* summarized that an antidoping program involving the cessation of AAS use led to improved fertility outcomes, as evidenced by increased live birth rates among former users. Over a 10-year period, fertility levels approached those of nonuser controls [25^▪▪^].

Cannabis is a widely used psychoactive substance. Its main active component, Δ9-tetrahydrocannabinol (THC), binds to receptors in the endocannabinoid system, which plays a regulatory role in the HPG axis, testosterone production, and spermatogenesis [17^▪▪^]. Chronic cannabis use has been associated with reduced sperm concentration, motility, and abnormal morphology [16^▪▪^,^17^^▪▪^]. Some studies suggest that these effects may be reversible, but long-term use alters hormone levels and is associated with spontaneous abortion in partners of male users [26^▪^].

Chronic opioid use has been shown to impair the HPG axis through μ-opioid receptor-mediated inhibition of gonadotropin-releasing hormone (GnRH), resulting in secondary testosterone deficiency. Opioids also increase oxidative stress and DNA fragmentation in sperm; long-term users often exhibit impaired chromatin condensation, increased apoptosis, and leukocytospermia [17^▪▪^].

Similarly, even commonly used over-the-counter medications such as paracetamol and new psychoactive substances have been associated with adverse effects on male fertility, including reduced semen volume and increased sperm DNA fragmentation. These findings underscore that both prescription and nonprescription drugs may negatively impact spermatogenesis [27^▪^].

## HEAT

Rising global temperatures raise concerns about testicular thermoregulation and fertility. Spermatogenesis is highly sensitive to temperature, so even a slight increase can impair sperm quality. A longitudinal study of over 54 000 men in Argentina by Verón *et al.* found that exposure to heat waves during sperm development significantly reduced sperm concentration, count, and morphology. Prolonged or repeated heat wave exposure was correlated with increasingly severe impairments. Surprisingly, vitality and motility sometimes increased, possibly as a compensatory mechanism or sampling artifact [28^▪^].

Verón *et al.* [28^▪^] concluded that relationship between heat exposure and semen quality greatly varies with heat intensity, duration, and timing of heat events relative to spermatogenesis.

## DIET

Diet also plays a foundational role in reproductive health. Numerous studies highlight the benefits of Mediterranean and Dietary Approaches to Stop Hypertension (DASH)-style diets – rich in fruits, vegetables, nuts, legumes, whole grains, and fish – on sperm concentration, motility, and morphology [26^▪^,^29^^▪▪^].

Meta-analyses indicate that men who adhere closely to healthy dietary patterns have significantly higher total sperm counts and better motility compared to those following Western-style diets rich in processed meats and trans fats [26^▪^]. Omega-3 fatty acids support sperm membrane fluidity and mitochondrial function, while saturated fats and cholesterol are associated with reduced sperm quality [30^▪^].

Protein source seems to matter as well – plant proteins and fish are beneficial, while high intakes of red and processed meat negatively affect semen parameters. Randomized controlled trials show that nut supplementation improves sperm vitality, motility, and morphology. Dairy findings are mixed – low-fat varieties may be protective, whereas full-fat dairy appears to be detrimental [26^▪^]. Conversely, excessive consumption of sugar-sweetened beverages and ultra-processed foods has been associated with a reduction in sperm quality and hormonal disruption [30^▪^].

Soy intake has raised concerns due to its phytoestrogen content; current meta-analyses show no significant adverse hormonal effects [26^▪^]. Micronutrients such as zinc, selenium, vitamins C and E, and folate – many of which have antioxidant properties – are critical for spermatogenesis and maintenance of sperm integrity [20^▪▪^]. Given their antioxidant role, these micronutrients are often investigated for their potential effects on reducing SDF. Indeed, intervention studies show that reductions in SDF after 3 months are similar regardless of treatment type, whether it is a combination of antioxidants and monotherapy (−4.27%, 95% CI: −6.11 to −2.43), antioxidants alone (−4.51%, 95% CI: −6.81 to −2.20), or monotherapy alone (−3.36%, CI: −4.44 to −2.28) – although overall, these interventions have only limited effects [23^▪^].

## POLLUTION

Environmental pollutants are increasingly recognized as significant contributors to impaired male reproductive health. Chronic exposure to air pollutants such as nitrogen dioxide, sulphur dioxide, carbon monoxide, and particulate matter (PM_2.5_, PM_10_) has been associated with reductions in sperm concentration, motility, morphology, and testicular volume. Several studies have reported that these pollutants induce oxidative stress and increase SDF [14^▪^,31^▪^].

The route of exposure also plays a significant role. Inhaled PM_2.5_ particles reach the bloodstream and testicular tissue, inducing systemic inflammation and autophagy at the blood–testis barrier through reactive oxygen species (ROS). Persistent exposure in polluted urban areas correlates with decreased testicular volume and sperm function [31^▪^].

Exposure to endocrine disruptors, such as phthalates and bisphenol A (BPA), has been linked to reduced testosterone levels, increased SDF levels, and deteriorated semen quality [14^▪^,32^▪^]. Although the effects of BPA on clinical fertility outcomes remain inconclusive, its influence on early puberty and hormonal imbalance is documented [14^▪^].

Persistent environmental contaminants such as polychlorinated dioxins, furans, and perfluoroalkyl and polyfluoroalkyl substances (PFAS) exhibit high toxicity; moreover, they have the potential to accumulate in the body [14^▪^,33^▪^]. These compounds, alongside pesticides and xenoestrogens, disrupt endocrine function by mimicking oestrogen activity or acting as antiandrogens, contributing to the development of testicular dysgenesis syndrome in male offspring, characterized by decreased fertility, hypogonadism, hypospadias, cryptorchidism, and testicular cancer [14^▪^]. Evidence also suggests that exposure to organochlorines through seafood consumption can impair sperm motility and chromatin integrity [16^▪▪^].

These observations highlight the need for public health interventions and further research into the reduction of pollution to safeguard fertility and reproductive health.

## CONCLUSION

This review highlights that modifiable lifestyle and environmental factors – including smoking, alcohol consumption, obesity, recreational drug use, poor diet, heat exposure, and pollution – significantly impair male fertility by disrupting hormonal regulation, increasing oxidative stress, and elevating SDF levels. Although conventional semen parameters are consistently affected, emerging evidence also underscores the role of epigenetic and transgenerational effects. Although partial reversibility is observed after cessation or intervention, these findings highlight the urgent need for targeted prevention strategies. By identifying and modifying these risk factors, clinicians can play a critical role in improving male reproductive health and reducing reliance on assisted reproductive techniques.

## Acknowledgements

*None*.

### Financial support and sponsorship

*Funding was provided by Semmelweis University.*

### Conflicts of interest

*There are no conflicts of interest.*

## REFERENCES AND RECOMMENDED READING

Papers of particular interest, published within the annual period of review, have been highlighted as:

▪ of special interest
▪▪ of outstanding interest
